# Transcriptional Regulation Associated with Subcutaneous Adipogenesis in Porcine *ACSL1* Gene

**DOI:** 10.3390/biom13071057

**Published:** 2023-06-29

**Authors:** Xiuqin Yang, Xiaohan Zhang, Zewei Yang, Qian Zhang, Wanjun Hao, Yu Pang, Dongjie Zhang, Di Liu

**Affiliations:** 1 College of Animal Science and Technology, Northeast Agricultural University, Harbin 150030, China; 2 Institute of Animal Husbandry, Heilongjiang Academy of Agricultural Sciences, Harbin 150086, China

**Keywords:** pig, ACSL1, transactivation, SNPs, linkage disequilibrium, adipogenesis, transcript variant

## Abstract

Long-chain acyl-CoA synthetase 1 (ACSL1) plays an important role in fatty acid metabolism and fat deposition. The transcription of the *ACSL1* gene is regulated specifically among cells and physiological processes, and transcriptional regulation of *ACSL1* in adipogenesis remains elusive. Here, we characterize transcription factors (TFs) associated with adipogenesis in the porcine *ACSL1* gene. CCAAT-enhancer binding protein (C/EBP)α, a well-known adipogenic marker, was found to enhance the expression of the *ACSL1* gene via binding two tandem motifs in the promoter. Further, we demonstrate that ACSL1 mediates C/EBPα effects on adipogenesis in preadipocytes cultured from subcutaneous fat tissue of pigs via gain- and loss-of-function analyses. The cAMP-response element binding protein, another TF involved in adipogenesis, was also identified in the regulation of *ACSL1* gene expression. Additionally, single nucleotide polymorphisms (SNPs) were screened in the promoter of *ACSL1* among four breeds including the Chinese indigenous Min, and Duroc, Berkshire, and Yorkshire pigs through sequencing of PCR products. Two tightly linked SNPs, −517G>T and −311T>G, were found exclusively in Min pigs. The haplotype mutation decreases promoter activity in PK-15 and ST cells, and in vivo the expression of *ACSL1*, illustrating a possible role in adipogenesis regulated by C/EBPα/ACSL1 axis. Additionally, a total of 24 alternative splicing transcripts were identified, indicating the complexity of alternative splicing in the *ACSL1* gene. The results will contribute to further revealing the regulatory mechanisms of *ACSL1* during adipogenesis and to the characterization of molecular markers for selection of fat deposition in pigs.

## 1. Introduction

Fat content is one of the main factors influencing growth and meat quality. Appropriate fat accretion contributes to improving the palatability and flavor of meat and is therefore favored by consumers, while excessive fat deposition brings potential hazard to human health. Additionally, the location of fat deposition is another issue troubling livestock producers. Subcutaneous and visceral fat are considered harmful to consumers, while intramuscular fat is associated positively with palatability and flavor [1,2]. Therefore, there are strong demands and intensive efforts for reducing the subcutaneous and visceral fat in production animals while simultaneously increasing intramuscular fat content. Therefore, this study provides an initial investigation in the essential role of fat-related genes and their regulatory network in adipogenesis.

Long-chain acyl-CoA synthetases (ACSLs) are essential in the activation of fatty acids (FAs) by converting them to fatty acid acyl-CoA esters [3,4]. The process is vital for both synthetic and oxidative pathways of cellular lipids including triacylglycerol (TAG), cholesterol esters, and phospholipids [5]. A total of five members were identified in the ACSL family. Each of these enzymes was encoded by a separate gene and had tissue-specific expression profiles, distinct substrate specificities, and different subcellular localizations [5,6]. ACSL1, responsible for the activation of FAs composed of 12–22 carbons, is the major member of the family in adipose tissue [7], indicating its importance in fat metabolism and deposition. Various studies have confirmed the role of ACSL1 in the synthesis of TAG, the main component of adipose tissue. For example, the ectopic expression of *ACSL1* promotes the accumulation of TAG by 12-fold in cardiomyocytes [8]; eliminating *ACSL1* decreases, and overexpressing *ACSL1* increases, the TAG synthesis in hepatocytes [9,10]; ACSL1 also facilitates FA uptake and TAG synthesis in mouse 3T3-L1 adipocytes [11], etc. The dysregulation of *ACSL1* is involved in obesity [12] and various diseases related to obesity including fatty liver disease [13,14] and diabetes [15,16]. ACSL1 was also identified as a transcription factor (TF) of genes essential for adipose development such as the family with sequence similarity 13 member A (*FAM13A*) [17,18].

Despite the increased knowledge of ACSL1′s role in fat deposition, the mechanisms underlying its transcriptional regulation in adipogenesis, including theTFs, are incompletely identified. Here, we report that porcine ACSL1 promotes adipogenesis via regulation by the CCAAT-enhancer binding protein (C/EBP)α, an adipogenic marker, and that a haplotype mutation in the promoter, frequently occurring in Min pigs, decreases the expression of the *ACSL1* gene. Pigs represent valuable biomedical models for studying human developmental processes and disease pathogenesis because of their high similarity to the human genome, anatomical structure and size, physiology, and immunology [19]. The results will contribute to further revealing the regulatory mechanisms of *ACSL1*, and the basis for manipulating its expression to maintain fat homeostasis. Additionally, the polymorphisms identified have great potential as molecular markers for selecting fat deposition in pig breeding.

## 2. Materials and Methods

### 2.1. Animals, Samples, and Nucleic Acid Isolation

Pigs were obtained from the Institute of Animal Husbandry, Heilongjiang Academy of Agricultural Sciences, Harbin, China. Tissue samples, used for cDNA cloning, including heart, spleen, muscle, lung, and liver, were collected from three 210-day-old Min pigs immediately after slaughter and snap frozen in nitrogen. A total of 51 individuals including 10 Yorkshire, 10 Duroc, 10 Berkshire, and 21 Min pigs were used for polymorphism analysis, and ear tissues were collected. Total RNA was isolated with TRIzol reagent (Invitrogen, Carlsbad, CA, USA). Genomic DNA was isolated with standard phenol-chloroform method without modification [20]. All procedures of animal treatment were strictly in accordance with the protocol of the Animal Care Committee of Northeast Agricultural University.

### 2.2. Plasmids and siRNA

The promoter of *ACSL1* was first characterized with online software packages Promo (https://alggen.lsi.upc.es/cgi-bin/promo_v3/promo/promoinit.cgi?dirDB=TF_8.3, accessed on 10 March 2021), Alibaba 2 (http://gene-regulation.com/pub/programs/alibaba2/, accessed on 10 March 2021), and Jaspar (https://jaspar.genereg.net/, accessed on 10 March 2021). The upstream sequences of porcine *ACSL1* spanning from −1649 to +62 bp were then amplified and inserted into pGL3 basic at enzyme sites *Xho* I and *Hin*d III to construct a reporter gene. Three plasmids containing truncated fragments, −1135~+62, −704~+62, −404~+62 bp, respectively, were then generated.

The complete coding sequences (CDS) of porcine *C/EBPα* were subcloned into pcDNA3.1+ vector to construct eukaryotic expression vector at enzyme sites *Kpn* I and *Eco*R I. Plasmids overexpressing cAMP-response element binding protein (CREB) were constructed previously [21]. Vectors carrying *ACSL1* CDS were constructed with pCMV-HA backbone at enzyme sites *Eco*R I and *Xho* I. All primers used in this study were designed with primer premier 5.0, synthesized by Beijing Genomics Institute (BGI: Beijing, China), and the sequences are listed in Table S1 except those used for mutagenesis. siRNA sequences (Table S2) were designed and synthesized by General Biol (Hefei, China).

### 2.3. Cell Line Culture and Transfection

PK-15 cells (RRID: CVCL_2160) were cultured in Dulbecco’s Modified Eagle’s Medium (DMEM)/Nutrient Mixture F-12 (F12). ST (RRID: CVCL_2204) and HEK-293T (RRID: CVCL_0063) cells were cultured with high-glucose DMEM. Both cultures were supplemented with 10% fetal bovine serum (FBS: Gibco, Carlsbad, CA, USA) and 1% penicillin-streptomycin (Invitrogen). The cells were incubated at 37 °C with 5% CO_2_. Transfection was conducted with Lipofectamine 2000 reagent (Invitrogen) based on the manufacturer’s instructions. In the case of induction of differentiation in porcine preadipocytes, the transfection was performed 24 h prior to induction, and to keep the knockdown efficiency of *ACSL1* during differentiation, the siRNA was transfected into cells every 48 h.

### 2.4. Mutagenesis

Site-directed mutagenesis was performed with overlap-extension PCR as described previously [22] to delete the putative binding sites of CREB and C/EBPα, respectively, in the promoter of *ACSL1*. Additionally, a point mutation was generated in the promoter to measure the effects of single nucleotide polymorphisms (SNPs) on the transcription of *ACSL1*. Fragments containing the desired mutation were inserted into pGL3 basic as described above. Primers and experiment schemes for overlap-extension PCR were shown in Tables S3 and S4.

### 2.5. Dual-Luciferase Reporter Gene Analysis

Each reporter gene was transiently transfected into PK-15/ST cells individually or together with vectors overexpressing C/EBPα or CREB. A *Renilla* luciferase reporter, pRL-TK, was used as an internal reference to avoid variations between groups. At 48 h after transfection, the cells were collected and luciferase activity was measured with a dual-luciferase reporter gene assay kit (Beyotime, Shanghai, China). The relative luciferase activity was calculated as a ratio of firefly to *Renilla* luciferase value.

### 2.6. Reverse Transcription and Real-Time Quantitative PCR

Reverse transcription (RT) was conducted with the HiScript III 1st Strand cDNA Synthesis Kit (+gDNA wiper) (Vazyme, Nanjing, China). Real-time quantitative PCR (qPCR) was performed with ChamQ Universal SYBR qPCR Master Mix (Vazyme) based on the manufacturer’s instructions, each with three replicates. Relative expression level of target gene was calculated using 2^−ΔΔCt^ method with *β-actin* as a reference. Primer sequences for qPCR are listed in Table S1.

### 2.7. Preadipocyte Culture, Differentiation, and Oil Red O Staining

Primary preadipocytes were isolated from subcutaneous fat tissues obtained from the newborn Min pigs. At each time point, three individuals were used for sample collection. The cells were cultured as described previously [21]. Briefly, fat tissues were filtered through 400-mesh filters after digested with 0.1% type I collagenase (Invitrogen). The resultant preadipocytes were cultured in DMEM/F12 supplemented with 10% FBS (Gibco) and 1% penicillin-streptomycin (Invitrogen). The medium was changed every two days.

The cells were induced to differentiate in DMEM/F12 medium containing 10% FBS, 1 µmol/L dexamethasone, 0.5 mmol/L 3-isobytyl-1-methylxanthine, and 5 µg/mL insulin. At 48 h post-induction, the medium was changed to DMEM/F12 containing 10% FBS and 5 µg/mL insulin to maintain the differentiation. The mature adipocytes were stained with Oil Red O kit (Leagene, Beijing, China), viewed under a light microscope and photographed (Carl Zeiss AG, Jena, Germany) to measure the cells morphologically. Cellular Oil Red O was isolated with isopropanol to quantify the lipid content with optical absorbance at 510 nm.

### 2.8. Western Blotting

At 48 h post-transfection, cells were collected for protein isolation using RIPA buffer (Beyotime, Shanghai, China) containing a protease inhibitor (Invitrogen). A total of 10~25 µg proteins were loaded on SDS-polyacrylamide gel and electrophoresed on 5% concentrated gel at 90 V for 30 min followed by separating gel at 120 V for 90 min. The proteins were then transferred to a polyvinyl difluoride membrane (Millipore, Shanghai, China). After blocking with 5% skimmed milk at 37 °C for 2 h, membranes were incubated at 4 °C overnight with anti-HA tag (1:5000 dilution; Abmart, Shanghai, China), -C/EBPα (1:1000 dilution; Abcam, Shanghai, China), -ACSL1 (1:1000 dilution; ABclonal, Wuhan, China), -GAPDH (1:5000 dilution; ABclonal, Wuhan, China), -β-tubulin (1:1000 dilution; Abcam), or -β-actin (1:2000 dilution; Abmart) primary antibodies. GAPDH, β-tubulin and β-actin were used as control in the experiments. The results were detected on UVP ChemStudio^TM^ PLUS touch produced by Analytik Jena US (Upland, CA, USA). Gray value analysis was conducted by ImageJ software (version 1.51j8) to calculate relative intensities of the bands.

### 2.9. Electrophoretic Mobility Shift Assay

Nuclear extracts were isolated from HEK-293T cells (RRID: CVCL_0063) with the nuclear extraction kit (Solarbio, Beijing, China). Electrophoretic mobility shift assay (EMSA) was performed with Chemiluminescent kit (Beyotime, Shanghai, China) according to the manufacturer’s instructions. The probe including biotin-labeled, unlabeled, and mutant ones for C/EBPα-specific binding were synthesized by General Biol (Hefei, China), and the sequences are listed in Table S5. After being annealed to double-stranded oligonucleotides, the probes were incubated with 20 µg nuclear extracts for 20 min at room temperature. In the competition group, unlabeled oligonucleotides were first incubated for 10 min before the addition of labelled probe. The mixture was electrophoresed on 6.5% polyacrylamide gel at 90 V for 1.5 h, and then transferred to a positively charged nylon membrane (Beyotime). The gels were visualized on Azure c300 Gel Imaging System (Azure Biosystems, Dublin, CA, USA).

### 2.10. Polymorphism Analysis

Fragments spanning −704~+62 bp were amplified with primers listed in Table S1 and the products were sequenced directly by BGI. The sequences were aligned with SnapGene (version 6.0.2) to screen SNPs. The SNP sites were confirmed by manual inspection of the sequence diagram with the Chromas software, which also made clear the genotypes of each animal. Linkage disequilibrium analysis and haplotype construction were performed with the SHEsis program (http://analysis.bio-x.cn/SHEsisMain.htm, accessed on 12 December 2022).

### 2.11. Alternative Splicing Transcript Identification

During construction of a eukaryotic expression plasmid of porcine *ACSL1* gene, the CDS was amplified from a cDNA mixture obtained from multiple tissues including heart, spleen, muscle, lung, and liver. The products were first cloned into a pMD18-T vector (Takara, Dalian, China) and transformed into DH5α. Colony PCR was performed with rTaq mix (Vazyme) and primer pair used for amplification of the complete CDS. The resultant products were detected in 1.5% agarose gel electrophoresis, and specific fragments differing in length from major CDS were screened. The corresponding colonies were subjected to sequencing by BGI to identify novel alternative splicing (AS) variants.

Additionally, full-length transcriptome analysis was performed in porcine muscles with PacBio (PB) sequencing previously [23]. To identify more AS variants, the PB data were screened and all the sequences aligned to *ACSL1* gene were collected. PB sequencing can ensure the completeness of the 3′ end, while there is deviance in the 5′ end. Thereafter, differences in the length of the 5′ end of the first exon were not considered as AS events to ensure accuracy, independent of any potential alternative transcript start site. Furthermore, the sequences without splicing or the 3′ end of the major transcript were filtered out. The novel variants were confirmed with RT-PCR using specific primers (Table S1), and the resultant products were subjected to sequencing by BGI.

### 2.12. Statistical Analysis

All experiments were conducted in triplicate. The data, shown as the mean ± standard deviation (SD), were from one representative experiment. Statistical analyses were performed with GraphPad Prism (version 9.5.1; GraphPad Software Inc., San Diego, CA, USA). The differences between two groups were analyzed with unpaired *t*-test, and those among multiple groups were analyzed with one-way ANOVA followed by Tukey’s multiple comparison test.

## 3. Results

### 3.1. C/EBPα Is Involved in the Transactivation of Porcine ACSL1

**Figure 1:**
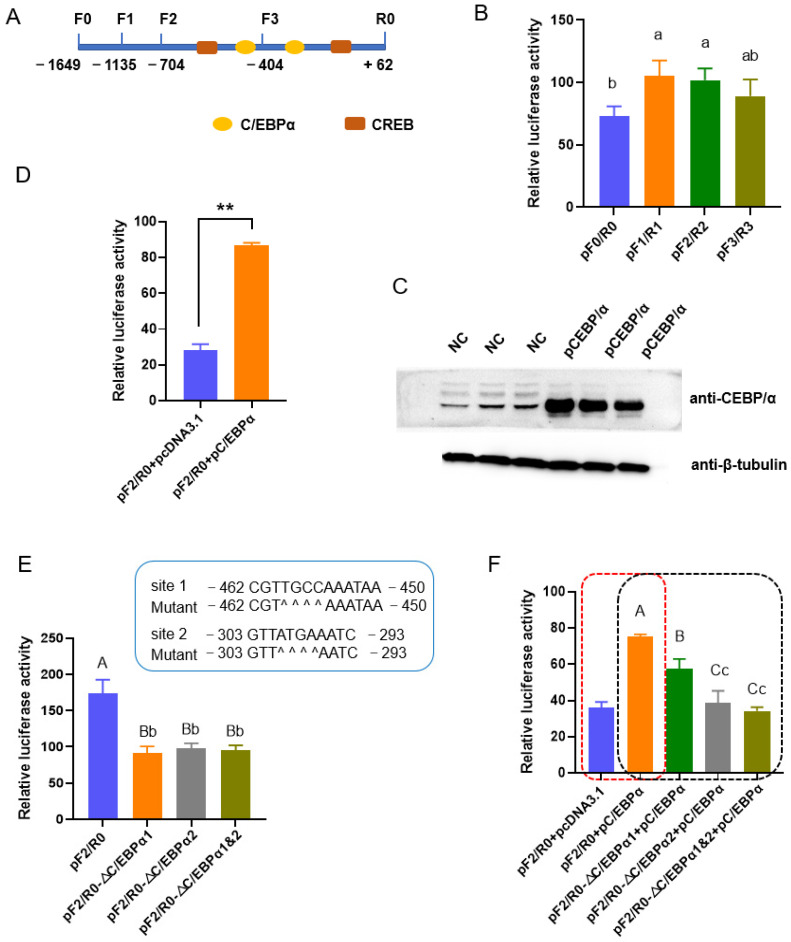

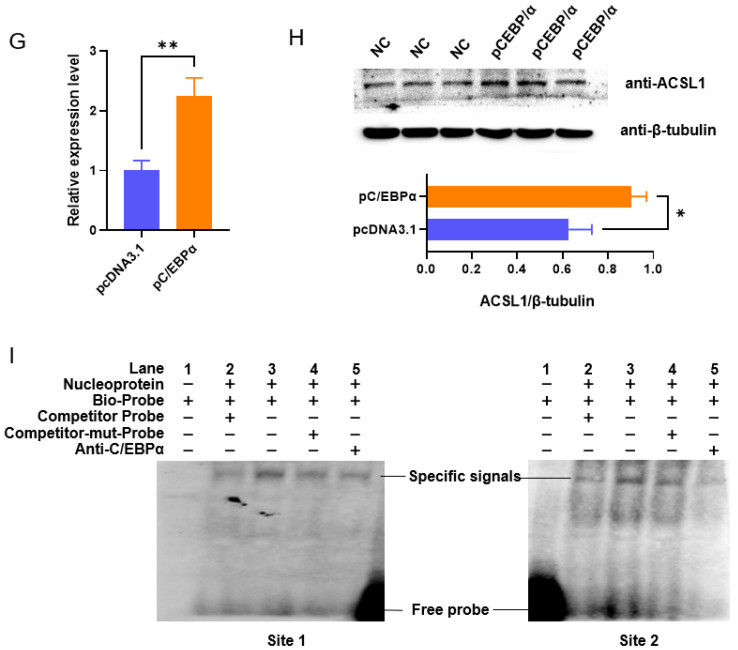
C/EBPα upregulates the expression of *ACSL1* through binding to two motifs in the promoter. (**A**) Illustration of putative binding sites of C/EBPα and CREB in the promoter of *ACSL1*. F and R indicate forward and reverse primer, respectively; (**B**) Promoter activity of the upstream sequence and a series of 5′ truncated fragments; (**C**) Overexpression efficiencies of vectors containing the coding sequences of porcine C/EBPα as revealed with western blotting; (**D**) Ectopic C/EBPα enhances the promoter activity; (**E**) Deletion of the binding sites of C/EBPα decreases the promoter activity. Scheme for deleting the two binding sites of C/EBPα is given above, and ^ indicates a nucleotide deletion; (**F**) Effects of C/EBPα on the promoter activity were abolished by deletion of the binding sites; (**G**) Ectopic C/EBPα increases the mRNA level of *ACSL1* gene; (**H**) Ectopic C/EBPα increases the protein level of *ACSL1* gene; (**I**) C/EBPα binds to the putative motifs as revealed by electrophoretic mobility shift assay. Differences identified: *, a, b, and c, *p* < 0.05; **, A, B, C, *p* < 0.01.

To investigate the transcriptional regulation of porcine *ACSL1* in adipogenesis, the upstream sequences were first analyzed with bioinformatic methods, and TFs associated with adipogenesis including C/EBPα and CREB were predicted (Figure 1A). A 1711 bp fragment of the 5′ flanking region (−1649~+62 bp) was then amplified and the promoter activity was confirmed with dual-luciferase reporter analysis. Truncated analysis showed that fragments spanning from −1135 to +62 bp have the highest promoter activity, and there is no obvious difference among fragments spanning −1135~+62, −704~+62 bp, and −404~+62 bp (*p* > 0.05). Fragments −1649~+62 bp have lower promoter activity than those spanning −1135~+62 (*p* < 0.05).

Two putative binding sites of C/EBPα, the well-known adipogenic marker, were mapped to −704~−404 and −404~+62 bp, respectively (Figure 1B). The overexpression of C/EBPα resulted in an increase in promoter activity of a reporter gene containing fragments −704~+62 bp (pF2/R0) (*p* < 0.01) in PK-15 cells (Figure 1C,D and Figure S1), indicating a positive role of C/EBPα on the expression of *ACSL1*. Subsequently, the putative binding sites of C/EBPα were deleted singly or simultaneously in pF2/R0. The deletion of any one of the C/EBPα sites decreased promoter activity in comparison to the wild type pF2/R0 (*p* < 0.01). The two mutant type pF2/R0 absent of the single C/EBPα site showed similar luciferase activity (*p* > 0.05), and no synergistic effects were found when the two sites were deleted simultaneously (*p* > 0.05) (Figure 1E). The promoting effects of exogenous C/EBPα were reversed when the binding sites were deleted, singly or simultaneously. It is interesting that site 2 inhibits the promoting effects of the C/EBPα much more strongly than that of site 1 (*p* < 0.01) (Figure 1F).

Furthermore, ectopic C/EBPα increases the expression of endogenous *ACSL1* at both mRNA (*p* < 0.01) and protein (*p* < 0.05) level (Figure 1G,H). EMSA showed that, in both binding sites, the DNA-protein complexes were formed clearly in the biotin-labeled probe (Lane 3) and mutation-competitor (Lane 4) groups, while the competitor probe groups (Lane 2) showed decreased intensity of the complex; the addition of a specific antibody against C/EBPα resulted in the weakness of DNA-protein complexes (Lane 5) (Figure 1I). These results indicated that C/EBPα could enhance the *ACSL1* expression through binding to the sites in the promoter.

### 3.2. CREB Regulates the Transcription of ACSL1

**Figure 2:**
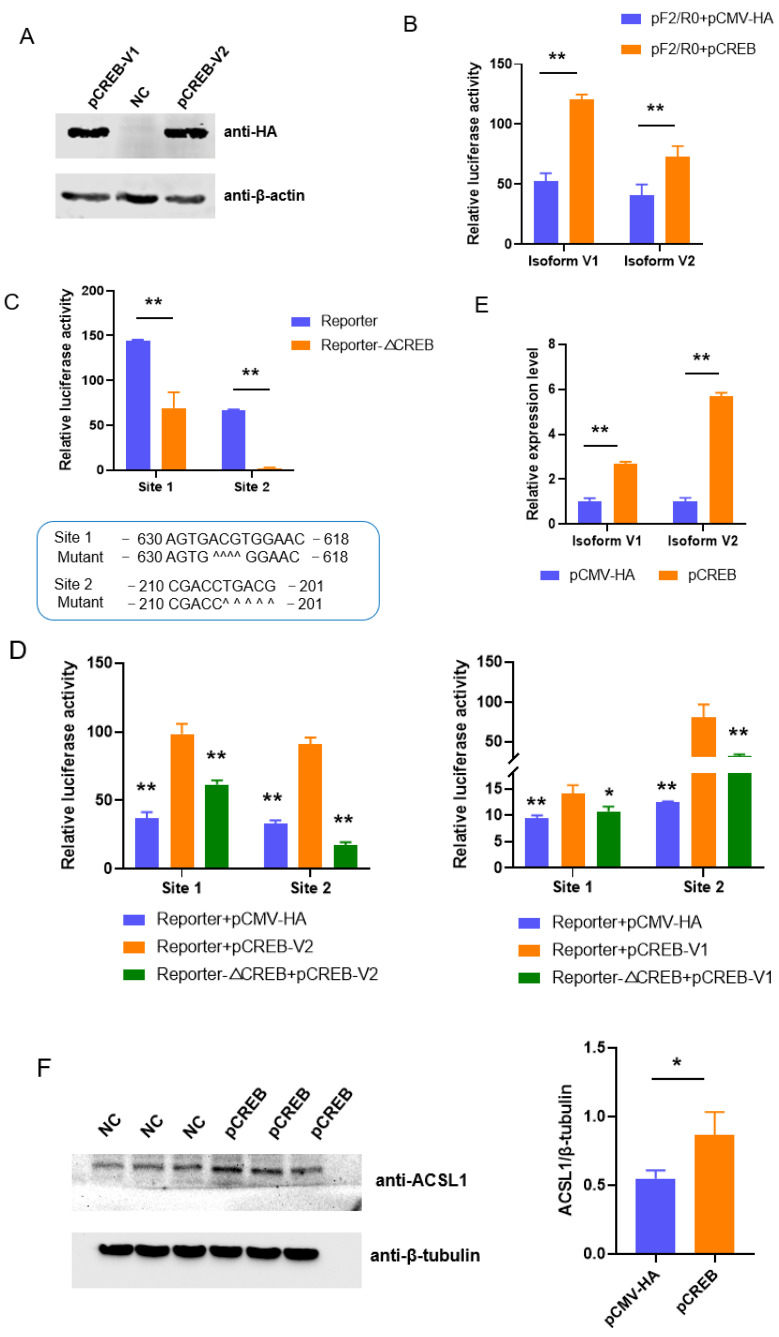
CREB upregulates the promoter activities of *ACSL1* through two binding sites. (**A**) Overexpression efficiencies of vectors containing the coding sequences of the two isoforms of porcine CREB; (**B**) Overexpression of both isoforms of CREB enhances promoter activity. (**C**) The putative binding sites of CREB positively regulate promoter activity. Scheme for deleting the binding sites of CREB is provided below, ^ indicates the nucleotide was deleted; (**D**) The promoting effects of CREB on promoter activity were reversed by inactivation of the binding sites. The values were compared with that in cells cotransfected with a wild type reporter gene and plasmids overexpressing CREB; (**E**) Ectopic CREB increases the mRNA level of *ACSL1*. (**F**) Ectopic CREB increases the protein level of *ACSL1*. * *p* < 0.05, ** *p* < 0.01.

Two binding sites of CREB were also predicted with a high reliability in the fragments spanning −704~+62 bp (Figure 1A). The effects of both major isoforms, V1 and V2, of CREB [22] on the promoter activity of *ACSL1* were first measured with an overexpression technique. Both isoforms can enhance the promoter activity of pF2/R0 (*p* < 0.01) (Figure 2A,B). Deleting any of the sites decreased promoter activity (*p* < 0.01) (Figure 2C), and the promoting effects of both isoforms on the promoters can be reversed by the mutation of the *cis*-elements (Figure 2D). Furthermore, overexpressed CREB enhances the transcription of endogenous *ACSL1* at both mRNA (*p* < 0.01) and protein (*p* < 0.05) level (Figure 2E,F).

### 3.3. ACSL1 Mediates the Effects of C/EBPα on Adipogenesis

**Figure 3:**
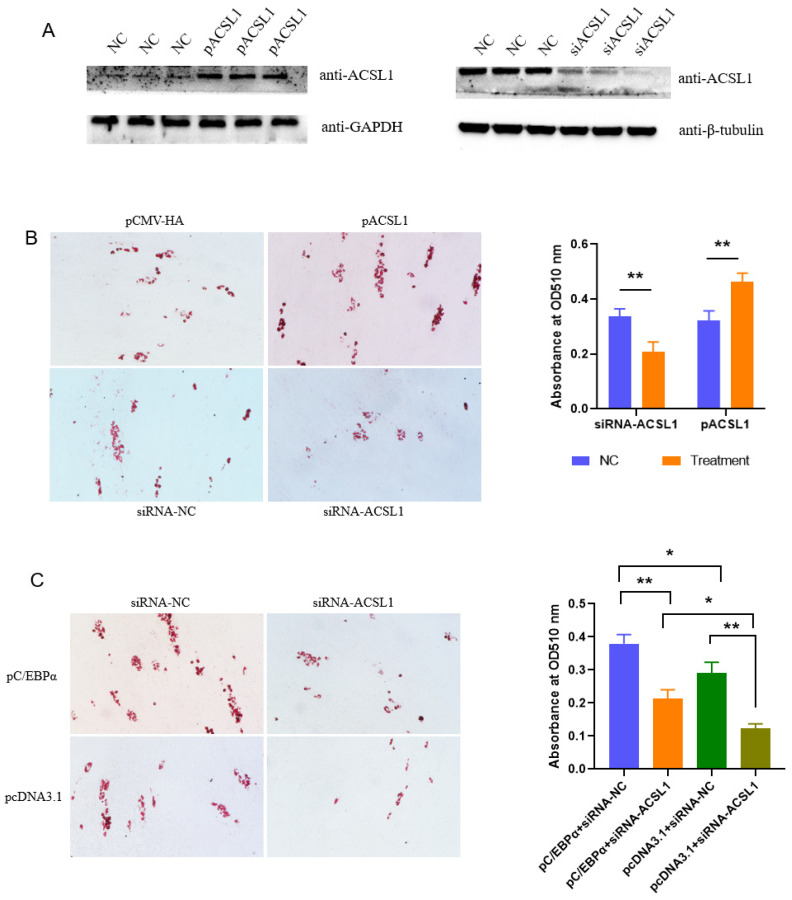
ACSL1 functions as a downstream gene of C/EBPα to promote adipogenesis. (**A**) Efficiencies of plasmids overexpressing C/EBPα and siRNA against C/EBPα; (**B**) Effects of ACSL1 on the differentiation of preadipocytes as revealed by Oil Red O staining and quantification of triglyceride; (**C**) ACSL1 mediates the effects of C/EBPα on adipogenesis. * *p* < 0.05, ** *p* < 0.01.

It is well known that C/EBPα is a key regulator of adipogenesis. To confirm whether ACSL1 functions during the adipogenesis regulated by C/EBPα, primary preadipocytes were first cultured from subcutaneous fat tissues of newborn pigs (Figure S2). siRNA-2031 can lower the expression of *ACSL1* to 20%, and thus was selected for further analysis. Both vectors and siRNA can work effectively at mRNA and protein level (Figure 3A and Figure S3). The overexpression of *ACSL1* promotes the differentiation of subcutaneous preadipocytes, while knockdown of *ACSL1* restrains the differentiation, as revealed by Oil Red O staining at 8-days post-induction. Consistently, TAG contents increased in preadipocytes overexpressing *ACSL1*, and decreased in cells knocking down *ACSL1* (Figure 3B). The results indicate that ACSL1 plays a positive role in porcine adipogenesis.

Rescue experiments showed that preadipocytes overexpressing *C/EBPα* formed more lipid droplets compared with those transfected with empty vector, while knocking down the expression of *ACSL1* reversed the promoting effects of C/EBPα on the preadipocyte differentiation. Consistently, TAG contents were increased by the ectopic expression of *C/EBPα*, and the promoting effect is decreased by inhibiting *ACSL1* expression (Figure 3C). These results indicate that *ACSL1* is a downstream gene in the adipogenesis regulated by C/EBPα.

### 3.4. SNPs Affect the Expression of ACSL1 in the Promoter

**Figure 4:**
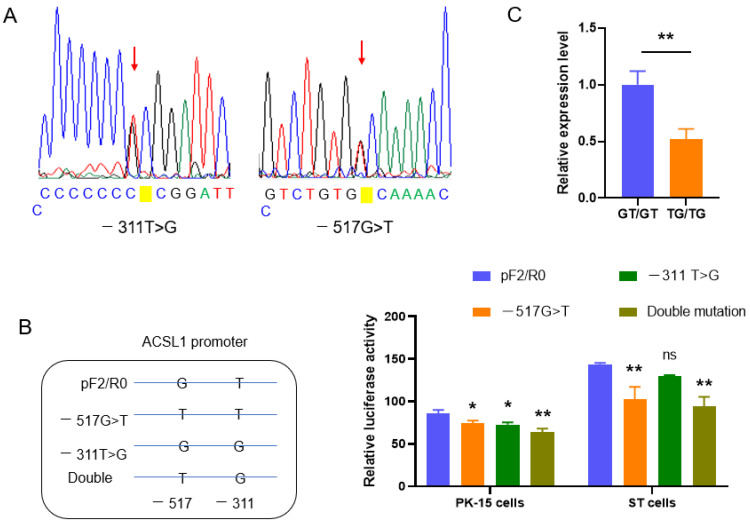
SNPs in the promoter are associated with the expression of *ACSL1*. (**A**) Sequencing diagram of SNPs exemplified with heterozygotes; (**B**) SNPs affect the promoter activity. The values were compared with that in cells transfected with wild type plasmid, pF2/R0; (**C**) Haplotype mutation decreases the expression of *ACSL1* in vivo. * *p* < 0.05, ** *p* < 0.01.

Two SNPs, −517G>T and −311T>G, were identified in the region spanning −704~+62 bp by direct sequencing of PCR products (Figure 4A). A total of 51 individuals were analyzed including 10 Yorkshire, 10 Berkshire, 10 Duroc, and 21 Min pigs. The SNPs were only found in Min pigs (Table 1). Among 30 individuals from the other three pig breeds detected, no polymorphisms were found at the two sites. The SNPs are in linkage disequilibrium with the parameter r^2^ = 0.909 and D’ = 1.0 as revealed by the online program SHEsis. They constitute three haplotypes, GT (50%), TG (47.6%), and GG (2.4%), in Min pigs studied.

To analyze the effects of the SNPs on the transcription of the *ACSL1* gene, −517G>T and −311T>G were mutated separately or simultaneously in the reporter gene pF2/R0. The single mutation of each SNP decreased promoter activity (*p* < 0.05), and synergistic effects were found in double mutation as revealed by dual-luciferase reporter analysis in both PK-15 and ST cells (Figure 4B). The effects of the SNPs on the expression of the *ACSL1* gene were further evaluated in vivo with homozygotes composed of the major haplotypes, GT or TG, and consistent results were obtained. The relative mRNA level was higher in homozygotes GT/GT than in TG/TG (*p* < 0.05) (Figure 4C). The results indicate that the haplotype mutation affects the expression of porcine *ACSL1*.

### 3.5. Porcine ACSL1 Is Rich in Alternative Splicing Transcripts

A total of four AS transcripts of the *ACSL1* gene, named RT-1~4, were identified through screening the positive clone with the PCR method during construction of the overexpression vector of *ACSL1*. Additionally, another 20 transcript variants, named PB-1~20, were identified through PB sequencing. PB-14, having unique sequences among all the transcripts, is selected for RT-PCR validation using a specific primer pair, and expected fragments were obtained. The sequences of RT-1~4 were deposited in the GenBank database with the accession Nos. OL449686~OL449689. The sequences of PB-1~20 are given in Table S6.

**Figure 5:**
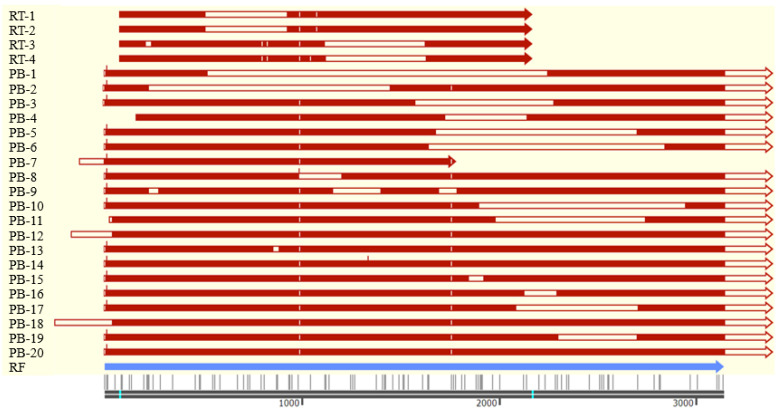
Schematic structure of transcript variants identified. RT, transcripts identified with RT-PCR method; PB, transcripts identified with PacBio sequencing; RF, reference sequence (GenBank No. NM_001167629.2). Open boxes indicate that there are deletions compared with the RF sequence. The red vertical lines indicate sequence insertion, while white vertical lines indicate single base substitution. The bright blue lines indicate the initiation and termination codon of RF sequence, respectively.

All the transcripts are mainly composed of exon sequences of *ACSL1* with various combinations (Table 2, Figure 5). Four of the five basic AS patterns, exon skipping (ES), intron retention (IR), alternative 3′ splice sites (A3SSs), and alternative 5′ splice sites (A5SSs), were found in the AS transcripts. A5SSs occurred with the highest frequencies, followed by A3SSs, ES, and IR among PB transcripts.

The 3′ end of the reference sequence (GenBank No. NM_001167629.2) was extended to 1596 bp through PB sequencing, while in the 5′ end, different lengths of the first exon were obtained and the longest one extended the sequence to 167 bp (Table 2, Figure 5). Additionally, the 5′ end of three transcripts, PB-11, 12 and 18, was located in intron 1. All PB transcripts contain sequences formed by alternative splice sites except for PB-8 and 12 formed by intron retention and/or exon skipping. Exons with the exception of exons 3, 4, 6, 7, and 12 harbor AS sites with the most in Exon 21. Additionally, a total of 29 specific AS sites including 16 A5SSs and 13 A3SSs were found in the PB transcripts. Most of the A5SSs exist in Exons 17 and 18, each with three. Seven of thirteen A3SSs exist in Exon 21, but no A5SSs were present in the region. Additionally, five different inner deletions were found in Exon 21, while no inner deletions were found in other exons. Thus, the most AS events were found in Exon 21 among all the PB transcripts.

## 4. Discussion

ACSL1 is essential for fat deposition through promoting the activation of long-chain FAs, which provide substrates for both the anabolism and catabolism of cellular lipids including TAG. The mRNA level of *ACSL1* is increased during pig adipogenesis [24,25]. However, the mechanisms underlying the upregulated expression of *ACSL1* in adipogenesis remain enigmatic, and SNPs with the potential for use for marker-assisted selection need to be identified. This study shows that the involvement of ACSL1 in the adipogenesis is regulated by C/EBPα, and a haplotype mutation in porcine *ACSL1* is associated with adipogenesis.

Several TFs have been involved in the transactivation of the *ACSL1* gene in humans and mice. Human *ACSL1* is transcriptionally regulated by NF-κB and carbohydrate response element binding protein in macrophages during hyperglycemia and inflammation [26]. Sterol regulatory element (SRE)-binding protein 2 activation induces hepatic *ACSL1* transcription through a SRE motif in the promoter [27]. In cattle TFs E2F1, Sp1, KLF15, and E2F4 drive the expression of *ACSL1* through directly binding to the promoter [28]. However, no studies were focused on the transcriptional activation of the porcine *ACSL1* gene. Here, TFs were first predicted with bioinformatic analysis in the promoter of procine *ACSL1* through which we found there are obvious differences in motif composition between the promoters of *ACSL1* in pig and cattle. The sequence alignment also showed that the identities were not very high, even in the motif sequences, in the proximal promoter region of the *ACSL1* gene among cattle, pigs, humans, and mice [28]. These indicate that the transcriptional regulation of *ACSL1* is breed-specific.

Various TFs were predicted in the 5′ flanking sequences of porcine *ACSL1*. This study focuses on TFs involved in adipogenesis such as C/EBPα and CREB, and no efforts were made to identify other factors influencing the promoter activity. C/EBPα, together with peroxisome proliferator-activator receptor-γ, are identified as the master adipogenic TFs [29]. C/EBPα is essential for lipid droplet formation by regulating the transcriptional activity of various fat-related genes including acetoacetyl-CoA synthetase [30], and fatty acid binding protein 4 [31]. The absence of C/EBPα inhibits the formation of white adipose tissue in vivo [32].

Despite the importance of ACSL1 in fat deposition, an intrinsic relationship between ACSL1 and C/EBPα has not been established. Here, we made clear that C/EBPα enhances the transcription of the *ACSL1* gene through two binding sites in the promoter. However, when double deletions were performed, promoter activity was enhanced. Through rechecking the sequences thoroughly in the region, a third binding site for C/EBPα was predicted at position of −146~−137 bp, downstream of site 2, which may be the reason why the double mutants maintained strong promoter activity. Whether the third motif is functional for C/EBPα remains to be identified. These two sites, located in −462~−450 and −303~−293 bp, respectively, are immediately adjacent to each other. There might be spatial interference to block the two sites being occupied simultaneously resulting in only one site being active in the wild type promoter, and thus no synergistic effects were found in double deletion mutants. Nevertheless we revealed that there are two binding sites of C/EBPα, located in −462~−450 and −303~−293 bp, respectively, in the promoter of the *ACSL1* gene.

Additionally, CREB, another positive regulator of porcine adipogenesis [21], was also shown to control the transcription of *ACSL1*. The two major isoforms, V1 and V2, have similar effects on the expression of *ACSL*, consistent with the previous results showing that only small differences existed in the expression profiles and transactivation activity between them [22]. Although we can not conclude that CREB functions through binding to the motif directly owing to the absence of data from EMSA or the chromatin immunoprecipitation method, it is clear that CREB is involved in the transcriptional regulation of *ACSL1*, and that the two motifs, located in −630~−618 and −210~−201 bp, respectively, are essential for the transactivation of ACSL1 by CREB. The fact that *ACSL1* is regulated transcriptionally by these adipogenesis-associated TFs, especially by the adipogenic marker, C/EBPα, indicates that it might function in the differentiation of preadipocytes to adipocytes.

Through gain- and loss-of-function assay, we first showed that ACSL1 promotes the preadipocyte differentiation, consistent with the results by Shan et al. [25], and then revealed that ACSL1 functions as a downstream gene of C/EBPα to regulate adipogenesis. Fat deposition is a dynamic process determined by adipogenesis, lipogenesis, lipolysis, and apoptosis. Numerous studies have revealed the role of ACSL1 in lipogenesis and lipolysis. The results obtained here provide new data for the regulation of ACSL1 in adipogenesis.

A total of five SNPs, located at promoter (−119T>C), exon 6 (173G>A), exon 14 (36C>T), exon 17 (46T>C), and 3′ untranslated region (UTR), respectively, were found in porcine *ACSL1* previously [33,34]. Three of them, −119T>C, exon 6 (173G>A), and exon 14 (36C>T), were only found in Tibet and Diannan small ear pigs, two Chinese indigenous pig breeds [34]. The SNPs, −517G>T and −311T>G, were first identified in pigs. Through genotyping in Min pigs, a Chinese indigenous fat-type pig breed, and another three lean-type pig breeds including Yorkshire, Berkshire, and Duroc, we found the SNPs only existed in Min pigs, suggesting a role of the SNPs in fat deposition.

As expected, the haplotype mutation was found to decrease the expression of porcine *ACSL1*; thus, it might have a role in adipogenesis regulated by the C/EBPα/ACSL1 axis and be a potential biomarker for the selection of fat deposition in pigs. SNPs in the *ACSL1* gene have been associated with trait performance. They were associated with milk production performance in buffalo [35] and Chinese Holstein Cows [36], body size traits in Dezhou donkeys [37], and polyunsaturated fatty acids in bovine skeletal muscle [38]. SNPs in the 3′ UTR of the human *ACSL1* gene influence its expression and thus results in poor clinical outcome in colon cancer patients [39]. To the best of our knowledge, this is the first report on the role of SNPs in the porcine *ACSL1* gene.

AS extensively occurs in eukaryotic genes. It was estimated that more than 95% of multi-exon genes can generate multiple transcripts through AS, increasing the complexity and diversity of proteome greatly [40,41]. AS is also involved in the regulation of gene expression through controlling the mRNA level of the major transcript [42]. Thus, AS is important for organisms and strictly regulated in tissues. AS transcripts of *ACSL1* have been identified in humans and sheep, but the number is limited. There are two AS variants in the human *ACSL1* gene resulting from mutually exclusive exons and ES, respectively, compared with the major one, leading to amino acids substitution or deletion in the inner of the polypeptide [43]. Alternative polyadenylation sites were found in the sheep *ACSL1* gene resulting in two AS variants: one had shorter 3′ UTR and the same polypeptide as the major one, while the other formed a truncated polypeptide owing to the presence of premature termination codon. The two isoforms of the sheep ACSL1 protein have a similar expression profile in tissues and differentiated preadipocytes [44]. These indicated that AS might function not only in the expression regulation of the *ACSL1* gene, but in the growth and development of the orgainsm.

Here, we identified a number of transcript variants of *ACSL1* with PB sequencing and RT-PCR methods. A total of 20 novel AS transcripts were obtained by PB sequencing in muscle tissues. PB sequencing can identify all AS transcripts in the sample theoretically, even those with humble expression, and thereafter exhausts the variants in muscle. However there is no overlap between PB transcripts and those obtained by the RT-PCR method. RT-PCR was perfomed in mixed tissues including muscle and viseral tissues to identify transcripts with differential length combined with agarose gel electrophoresis. The sensitivity of agarose gel electrophoresis depends on the difference of length and the expression level of mRNA. Those transcripts with similar length or humble expression can not be found by this method. The results indicate that the AS is differentially occurred in tissues, and more variants remain to be identified in the *ACSL1* gene. Compared to those obtained in humans and sheep, most AS variants in pigs have out-of frame mutation and can not encode polypeptides with similar roles to the major one as revealed by motif composition, suggesting a main role of AS in regulating the expression level of the major one. Efforts will be made to clarify the role of AS in pigs in future. Anyway, we made clear here that porcine *ACSL1* is rich in AS and more variants remain to be found.

## 5. Conclusions

In conclusion, we demonstrate that C/EBPα and CREB, two TFs involved in adipogenesis, are regulators of *ACSL1* gene expression, and that C/EBPα regulates the expression of the *ACSL1* gene through two binding motifs. ACSL1 mediates the promoting effects of C/EBPα on the adipogenesis. The haplotype mutation formed by two tightly linked SNPs, −517G>T and −311T>G, frequently occurred in Min pigs, a Chinese indigenous breed, resulting in a decrease in promoter activity and the in vivo expression of *ACSL1.* The influence on adipogenesis regulated by the C/EBPα/ACSL1 axis can readily serve as a biomarker for selecting traits associated with fat deposition.

## Figures and Tables

**Table 1:** SNPs identified in Min pigs.

Locus	Allele Frequency (%)		Genotype Frequency (%)		
	G	T	GG	GT	TT
−517G>T	50	50	23.81 (5) *	52.38 (11)	23.81 (5)
−311T>G	47.62	52.38	23.81 (5)	57.14 (12)	19.05 (4)

* The number of individuals are given in the parentheses.

**Table 2:** Alternative splicing transcripts of porcine *ACSL1.*

	Length	Sequence Composition
RF	3133	E1(124 ^$^+43)+E2(227)+E3(115)+E4(65)+E5(102)+E6(100)+E7(179)+E8(33)+E9(52)+E10(74)+E11(78)+E12(135)+E13(135)+E14(96)+E15(73)+E16(89)+E17(117)+E18(144)+E19(102)+E20(72)+E21(1102+494 ^$^)
RT-1	1688	E2 +E3~4+E5[1~63]+E10[9~74]+E11~21
RT-2	325	E2[30~220]+E21[8~141]
RT-3	1566	E2[30~163]&[190~220]+E3~11+E12[1~48]+E17[29~117]+E18~21
RT-4	1594	E2+E3~11+E12[1~53]+ E17[32~117]+E18~21
PB-1	1916	E1~4+E5[1~72bp]+E21[213~1596]
PB-2	2419	E1+E2[1~178]+E15[10~73]+E16~21
PB-3	2942	E1~15+E16[1~68]+E21[245~1596]
PB-4	3060	E2[114~227]+E3~13+E18[1~15]+E21[109~1596]
PB-5	2619	E1~16+E17[1~83]+E21[667~1596]
PB-6	2439	E1~16+E17[1~43]+E21[807~1596]
PB-7	1919	E1~17+E18(1~72)
PB-8	3421	E1~10+E13~21
PB-9	3263	E1+E2[1~178]+E3~11+E12[1~90]+E14[61~96]+E15~16+E17[1~103]+E18[74~144]+E19~21
PB-10	2593	E1~18+E19[1~45]+E21[916~1596]
PB-11	2855	I1(27)+E2~19+E20[1~25]+E21[712~1596]
PB-12	3802	I1(216)+E2~21
PB-13	3613	E1~7+E8[1~23]+E9[17~52]+E10~21
PB-14	3648	E1~9+E10[10~74]+E11~13+I13(9)+E14~21
PB-15	3567	E1~17+E18[1~137]+E19[65~102]+E20~21
PB-16	3482	E1~20+E21[1~101]&[263~1596]
PB-17	3024	E1~20+E21[1~59]&[676~1596]
PB-18	3786	I1(299)+E2~20+E21[1~1363]&[1463~1596]
PB-19	3240	E1~20+E21[1~266]&[667~1596]
PB-20	3538	E1~20+E21[1~1429]&[1525~1596]

RF, reference sequence (GenBank No. NM_001167629.2). RT, transcripts identified with RT-PCR method with primers amplifying the complete coding sequences, spanning from the 30th bp of Exon 2 to the 141st bp of Exon 21, of RF. PB, transcripts identified by PacBio sequencing. E, Exon; I, Intron. Figures in parentheses indicate length of the exon/intron, while those in brackets indicating nucleotide sequences spanning the region of the exon are included in the transcripts. Those exons without brackets indicate the sequences are the same as that in RF. ^$^ The 124 and 494 bp is extended by PB sequencing in the 5′ and 3′ end, respectively.

## Data Availability

The relevant data are available from the corresponding author upon reasonable request provided along with the manuscript as Supplementary Files.

## Supplementary Materials

The following supporting information can be downloaded at: https://www.mdpi.com/article/10.3390/biom13071057/s1, Table S1: Primers used for fragment amplification; Table S2: siRNA sequence synthesized; Table S3: Primers used for site-directed mutagenesis; Table S4: Schemes for site-directed mutagenesis using overlap-extension PCR; Table S5: Oligonucleotides used for electrophoretic mobility shift assay; Table S6: The sequences of PB-1~20. Figure S1: Characterization of the efficiencies of overexpression vector of C/EBPα; Figure S2: Characterization of preadipocytes cultured; Figure S3: Efficiencies of overexpression vector and siRNA of ACSL1.
