# Supplementary material for: Transcriptional Regulation Associated with Subcutaneous Adipogenesis in Porcine ACSL1 Gene
Source: Biomolecules. 2023 Jun 29;13(7):1057. doi: 10.3390/biom13071057 (PMC10377008; doi:10.3390/biom13071057)
Supplement: Supplementary file 1 [file biomolecules-13-01057-s001.zip › Figures S1-S3.pdf]

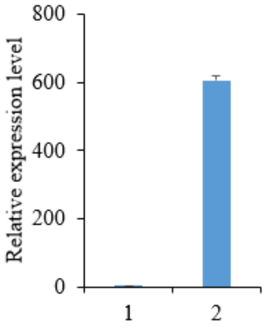

Figure S1. Characterization of the efficiencies of overexpression vector of C/EBPα

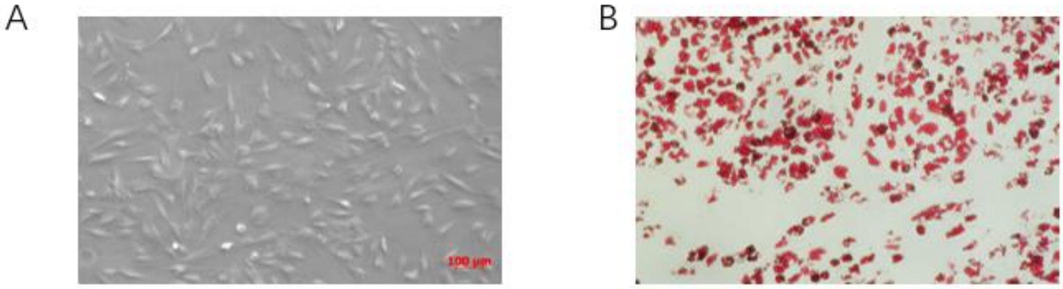

Figure S2. Characterization of preadipocytes cultured with morphological observation under electron microscope (A) and Oil Red O staining (B).

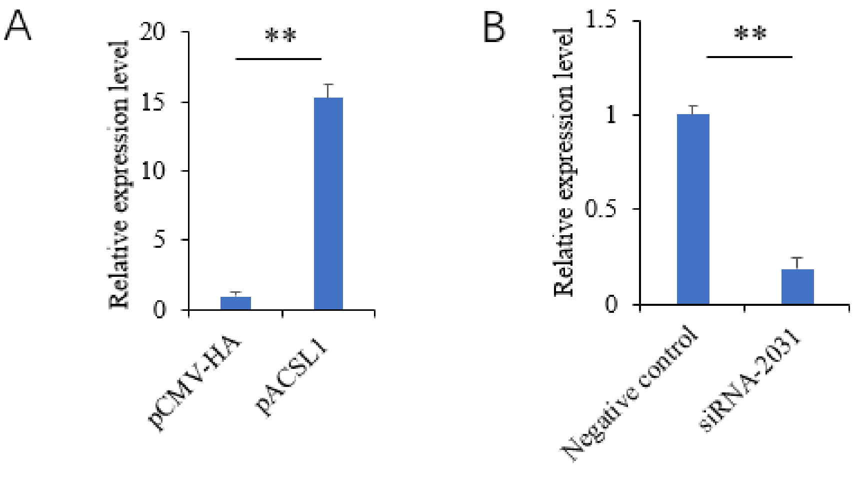

Figure S3. Efficiencies of overexpression vector (A) and siRNA of ACSL1 (B)
