# Supplementary material for: Transcriptional Regulation Associated with Subcutaneous Adipogenesis in Porcine ACSL1 Gene
Source: Biomolecules. 2023 Jun 29;13(7):1057. doi: 10.3390/biom13071057 (PMC10377008; doi:10.3390/biom13071057)
Supplement: Supplementary file 1 [file biomolecules-13-01057-s001.zip › Table S1-S5 .pdf]

Table S1 Primers used for fragment amplification

Sequence (5′-3′)	Purpose
F: CCGCTCGAGTCACGCAGCGAAAATGATCCG<br>R: CCCAAGCTTAGCACTCACCTCCTCCGCAG	Amplification of – 1649 to + 62 bp of ACSL1
F: GTAGTGAGCGATTGTTCAGCGTTTG<br>R: GCGAGAGGCAAGAAAGAGATCAGAG	qPCR for ACSL1
F: CATCACCATCGGCAACGA<br>R: GCGTAGAGGTCCTTCCTGATGT	qPCR for β-actin
F: TAGACAAGAACAGCAACGAG<br>R: ACCTTCTGTTGAGTCTCCACG	qPCR for C/EBPα
F: TAGCCCTCAAAACCGCACC<br>R: AGCACTCACCTCCTCCGCAG	Polymorphism analysis
F: CATCAACAAAGGCGAACTC<br>R: CTTTTCCTCCCAGGCTTGA	RT-PCR validation of transcript variants identified by PacBio-sequencing

Enzyme sites were underlined. F, forward; R, reverse; the same as below.

Table S2 siRNA sequence synthesized

Name	Sequence
siRNA-800	CAGCGGAAGCUCUGGAUAATT<br>UUAUCCAGAGCUUCCGCUGTT
siRNA-1652	GGGUGGAGAUCAUGAGCAUTT<br>AUGCUCAUGAUCUCCACCCTT
siRNA-2031	GGUCAGAGGUAUUAGUCUUTT<br>AAGACUAAUACCUCCUGACCTT
siRNA-NC	UUCUCCGAACGUGUCACGUTT<br>ACGUGACACGUUCGGAGAATT

Table S3 Primers used for site-directed mutagenesis

Name	Sequence (5′-3′)
F0<br>R0	The same as that for amplification of – 1649 to + 62 bp of ACSL1
C/EBPα-1MF	CTGCACATCGTAAATAAAATAGGAA
C/EBPα-1MR	CTATTTTATTTACGATGTGCAGGAC
C/EBPα-2MF	CGGATTCGTTAATCCCGGGACA
C/EBPα-2MR	TGTCCCGGGATTAACGAATCCG
CREB-1MF	CTGCCATTAGTTGGAACCAGTGCG
CREB-1MR	ACTGGTTCCAACTAATGGCAGTGTT
CREB-2MF	CGCGCGACCAAACGGGAAGGC
CREB-2MR	GCCTTCCCGTTTGGTCGCGCGC
− 517G>T-MF	CAGGTCTGTGTCAAAACCCCAC
− 517G>T-MR	GTGGGGTTTTGACACAGACCTG
− 311T>G-MF	GCCCCCCCGCGGATTCGTTAT
− 311T>G-MR	AACGAATCCGCGGGGGGGCA

Table S4 Schemes for site-directed mutagenesis using overlap-extension PCR

Sites	Reaction A	Reaction B	Templates for A and B	Reaction C	Templates for C
C/EBPα-1	F2/C/EBPα-1MR	C/EBPα-1MF/R0	pF2/R0	F2/R0	Products of reactions A and B
C/EBPα-2	F2/C/EBPα-2MR	C/EBPα-2MF/R0	pF2/R0	F2/R0	
C/EBPα-double	F2/C/EBPα-2MR	C/EBPα-2MF/R0	pF2/R0*	F2/R0	
CREB-1	F2/CREB-1MR	CREB-1MF/R0	pF2/R0	F2/R0	
CREB-2	F3/CREB-1MR	CREB-1MF/R0	pF3/R0	F3/R0	
−517G>T	F2/−517G>T-MR	−517G>T-MF/R0	pF2/R0	F2/R0	
−311T>G	F2/−311T>G -MR	−311T>G -MF/R0	pF2/R0	F2/R0	
SNP-double	F2/−311T>G -MR	−311T>G -MF/R0	pF2/R0**	F2/R0	

Plasmids pF2/R0* containing mutated sites of C/EBPα-1; pF2/R0** containing mutated sites of SNP −517G>T.

Table S5 Oligonucleotides used for electrophoretic mobility shift assay

	Name	Sequence (5′-3′)
**Site 1**	Bio-probe-F	bio-GACAGATATAGATTTGGACCTAGCC
	Bio-probe-R	bio-GGCTAGGTCCAAATCTATATCTGTC
	Competitor-F	GACAGATATAGATTTGGACCTAGCC
	Competitor-R	GGCTAGGTCCAAATCTATATCTGTC
	Competitor-mut-F	GACAGATATAGGCCCTTACCTAGCC
	Competitor-mut-R	GGCTAGGTTTCCCGCTATATCTGTC
**Site 2**	Bio-probe-F	bio-AGGCTTCCATTAGGAAATTCGTTCC
	Bio-probe-R	bio-GGAACGAATTTCCTAATGGAAGCCT
	Competitor-F	AGGCTTCCATTAGGAAATTCGTTCC
	Competitor-R	GGAACGAATTTCCTAATGGAAGCCT
	Competitor-mut-F	AGGCTTCCATTAGACCCGTCGTTCC
	Competitor-mut-R	GGAACGAGCCCACTAATGGAAGCCT
